# Tell Me, What Do You See?—Interpretable Classification of Wiring Harness Branches with Deep Neural Networks

**DOI:** 10.3390/s21134327

**Published:** 2021-06-24

**Authors:** Piotr Kicki, Michał Bednarek, Paweł Lembicz, Grzegorz Mierzwiak, Amadeusz Szymko, Marek Kraft, Krzysztof Walas

**Affiliations:** 1 Institute of Robotics and Machine Intelligence, Poznań University of Technology, Piotrowo 3A, 60-965 Poznań, Poland; michal.bednarek@put.poznan.pl (M.B.); amadeusz.szymko@put.poznan.pl (A.S.); marek.kraft@put.poznan.pl (M.K.); krzysztof.walas@put.poznan.pl (K.W.); 2 Volkswagen Poznań Sp. z o.o., ul. Warszawska 349, 61-060 Poznań, Poland; pawel.lembicz@vw-poznan.pl (P.L.); Grzegorz.Mierzwiak@vw-poznan.pl (G.M.)

**Keywords:** machine vision, deformable linear objects, neural networks, robot learning, computer vision for manufacturing

## Abstract

In the context of the robotisation of industrial operations related to manipulating deformable linear objects, there is a need for sophisticated machine vision systems, which could classify the wiring harness branches and provide information on where to put them in the assembly process. However, industrial applications require the interpretability of the machine learning system predictions, as the user wants to know the underlying reason for the decision made by the system. We propose several different neural network architectures that are tested on our novel dataset to address this issue. We conducted various experiments to assess the influence of modality, data fusion type, and the impact of data augmentation and pretraining. The outcome of the network is evaluated in terms of the performance and is also equipped with saliency maps, which allow the user to gain in-depth insight into the classifier’s operation, including a way of explaining the responses of the deep neural network and making system predictions interpretable by humans.

## 1. Introduction

In industrial robotics, much emphasis is placed on the automation of processes currently executed by humans. That demand leads to an improvement in the accuracy of task execution and a reduction of the time needed for it. That, in turn, inevitably increases production performance and results in many gains in terms of financial and human resources in a production facility. Most tasks requiring dexterous manipulation are solvable using industrial robots performing repetitive movements with position control. However, the challenge arises when a station performs a task that requires the autonomous perception of an environment. One can still observe a significant gap between the efficiency in the sensory systems of humans and that in industrial robots, which creates the need to hire people for tedious and repetitive work. For example, a computer vision system would play a significant role in an inspection task, working as a supervisor that processes an input image and applies an appropriate control for the robotic manipulator to, for example, disentangle a wire or localise functional parts of the harness during the wiring process in a car.

Because of the growing demand for electronics in cars from early 1990, the automatic inspection of a car wiring harness became a significant challenge in the robotics community. Contrary to traditional image processing methods, to solve complicated problems of a perceptual nature, in recent years, the research community’s state-of-the-art approaches have been based on machine learning, such as for object recognition [1,2], or robot control for manipulation [3]. However, there are very few industrial-oriented works that one could utilise in existing production facilities. The problem is that machine learning systems are often treated as black boxes [4], which is an undesirable situation for industrial applications.

Therefore, we provided a cabling inspection system based on visual data and deep neural networks. The system is intended to be used as the guiding tool for robotic manipulation. Moreover, we created a dataset (available at https://chmura.put.poznan.pl/s/XIiZgNfCMCuj1bY/download (accessed on 22 June 2021)) for training machine learning algorithms in the task of classification of branches of the car wiring harness. Additionally, to address the interpretability of machine learning system predictions, we implemented a mechanism for the visualisation of input image regions that are crucial for the executed task. As a result, we obtain heatmaps in the same shape as the input images, where brighter regions illustrate critical parts, which will guide the robotic manipulation process. We refer to the maps as *saliency maps*.

In our paper, first, we will preview the related work. Afterwards, we will provide a comprehensive description of the methods and datasets utilised in our experiments. Next, we will present the outcome of our experiments. Then, we will conduct a discussion on the results. Finally, we will provide the concluding remarks.

## 2. Related work

In this section, we provide a comprehensive state-of-the-art review on wiring analysis and image region-sensitive methods in convolutional neural networks.

**Wiring analysis**. Automatic disassembly of electronic devices was tackled in [5], which requires a good understanding of the wiring of a machine. The authors of [6] performed a complete wire analysis that yields the component detection, positioning and length measurements for effective robotic manipulation. In [7], a preliminary study of connector detection was conducted. Another inspection method was proposed in [8], where the image processing method for tangle detection was proposed. Trying to meet the manufacturers’ requirements, the authors of [9] propose a self-learning algorithm for insulation inspection, while [10] presents a method for error finding in the wiring harness. Due to the lack of commercial solutions in the field of automatic wiring analysis, the authors of [11] propose a method for the automatic execution of cable insertion into commercial electro-mechanical components. A switchgear wiring process was the subject of research in [12,13]. The solution includes the design of hardware and software and a setup with industrial robotic manipulators. Moreover, [14] presented the design of a specialised tactile sensor for the estimation of the position and orientation of a wire. The flexibility of cables is a challenging problem during the insertion task. In [15], the authors proposed a vision-based method for calculating the force acting on a wire from a stereo vision camera. Whereas, in [16] a method for the estimation of the bend of a cable-like object was proposed. Additionally, in [17], a method for inserting a flexible cord was presented while supervising its shape with a vision system. Another effective implementation of wire insertion into a switchgear assembly for a robot manipulator was proposed in [18]. The system utilises both haptic and vision sensors to execute the task. In [19], the authors also used both haptic and visual feedback to manipulate the deformable linear object bimanually. Whereas, in [20] a vision-based only system was used to perform deformable object manipulation.

**Saliency maps**. Producing coarse localisation maps in deep neural networks that highlight input image regions essential for a task was also tackled in [21]. The authors based their research on [22], in which a method for mapping unseen objects into the dictionary of known concepts to learn classifiers for novel classes was presented. Based on this, the Human Importance-aware Network was presented in [23], where a deep neural network was encouraged to be sensitive to the same input regions as humans, which was effectively visualised. In [24], the authors investigated masking-based methods for saliency maps generation, while in [25], a top-down excitation backpropagation technique was proposed. Semantic segmentation, based on color and depth images that generate object-like region proposals before classification, was proposed in [26]. The authors of [27] presented a new method, called *class activation mapping (CAM),* for finding discriminating features in the image region without being trained on them. In our research, we used both the CAM algorithm, as well as its adaptation to a class-agnostic setup, to determine which parts of the image attract the classifier’s attention in general, without conditioning on the specific class. To do so, we do not multiply the accumulated feature maps gathered right before the *Global Average Pooling Layer* by the class-feature weights in the last layer. By neglecting conditioning on the specific class, we identify whether the classifier uses relevant data (parts of the image) to perform classification in general.

## 3. Materials and Methods

### 3.1. Industrial Context

**Figure 1:**
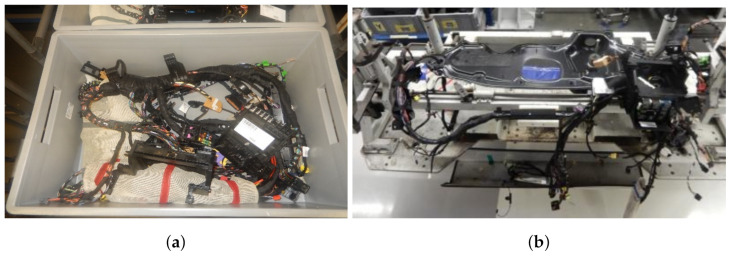
Wiring harness at the production line: (**a**) in the box, (**b**) at the mounting plate.

The work presented in this paper is related to the wiring harness mounting in the cockpit of the VW Caddy produced at the factory in Poznan, Poland. The task in general consists of taking the wiring harness from the box (Figure 1a) and putting it on the mounting plate (Figure 1b). The research described here, the goal of which is to identify the branches of a harness, is part of a more extensive system whose task is to provide the robot with information about the most convenient grasping points for performing the manipulation task. These points are, however, not fixed as the harness deformation causes them to move. Therefore, a perception system is required. In our research, we propose a learning-based solution for the identification of the wiring harness branches.

### 3.2. Dataset

Using neural networks to recognise the functional parts of a wiring harness requires data to train neural network models. Our paper introduces a novel dataset of RGBD images of wiring harness branches, which are parts of a wiring harness of a *Volkswagen Caddy* cockpit.

**Figure 2:**
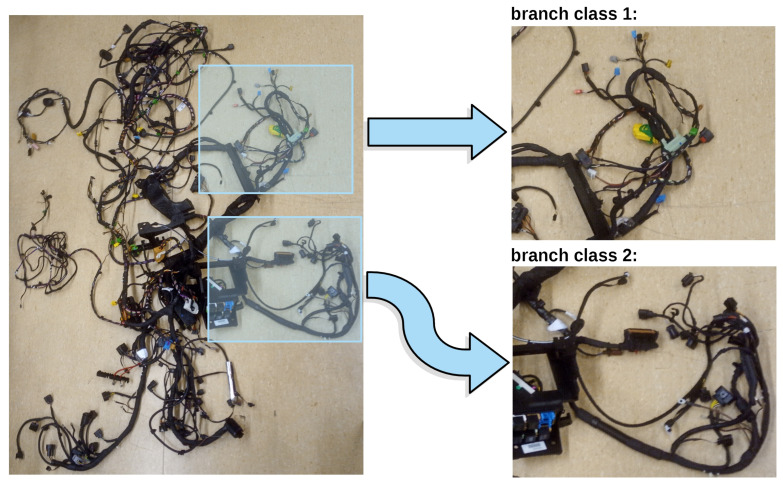
Sample image of the wiring harness. Example branches of two classes are highlighted in the source image and shown in detail on the right.

**Figure 3:**
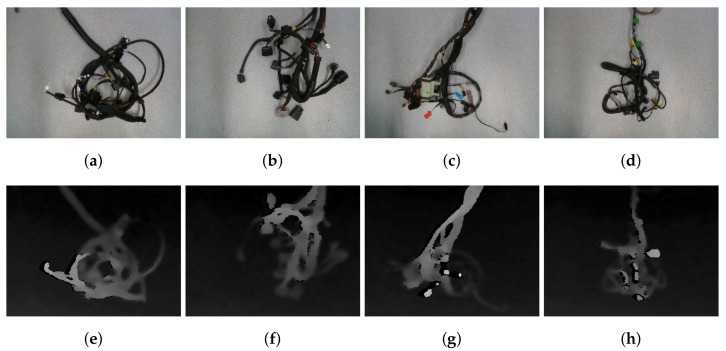
Samples from the dataset: (**a**–**d**) are the RGB images, (**e**–**h**) associated with depth images. Each pair comes from the different class: (**a**,**e**) branch 1, (**b**,**f**) branch 2, (**c**,**g**) branch 3, (**d**,**h**) branch 4.

Images from the dataset were assigned to one of four classes, which denote different wiring harness branches. By branch, we mean the functional part of the wiring harness, which in the process of the car cockpit assembly needs to be mounted in a specific place in the cockpit as it has to connect some specific car electrical appliances. Each branch has its specific function; thus, they must be recognised in order to put them in the right place to mount them properly. In our paper, we refer to these branches as *branches* 1, 2, 3, 4. A sample image of the whole wiring harness, with example branches highlighted, is shown in Figure 2. In Figure 3, we present the exemplary elements of the dataset; one for each category.

RGBD images in the dataset were gathered using the *Realsense D435 Depth Camera*, which was mounted firmly 50 cm above the ground level, where the wiring harness was located. The image background remained the same throughout the whole experiment, as did the lighting conditions, due to the artificial light source (fluorescent lamps). While creating the dataset, the wiring harness was rearranged between consecutive frames to create different harness arrangements. This was done to imitate possible states in which the machine vision system can see the harness in a real-life application. The dataset consists of 600 aligned RGB and depth images at a resolution of 640 × 480 pixels, split equally into four classes. Considering the ranges of the depth data obtained from the sensor, we preprocessed it to expose objects from the background maximally. In Figure 3, we present the exemplary elements of the dataset. Samples coming from different categories differ in terms of the topology of the branch, its geometry and the color and shape of the electrical connectors. However, some connectors can be found in more than one type of branch.

The dataset was split into three folds (I, II and III), each containing 200 samples equally distributed between four classes. Data in each fold come from a different sequence of the data gathering procedure. The motivation for such a split is that we can perform three-fold cross-validation using those folds with the data coming from a slightly different distribution. For each fold, the wiring harness was shaken, squeezed and bent to achieve a different state.

**Preprocessing**. To save the 16-bit depth values to the 8-bit format without losing the resolution, the following preprocessing step was performed. Taking into account that the wiring harness lay on the ground and did not stick out further than 25.5 cm, we could map the 16-bit values to the range of 8-bit (256 depth values) with the resolution at the level of 1 mm (the resolution of the depth gathered with the *Realsense D435* camera). Henceforth, we performed a few operations: (i) find a maximal depth value (distance to the background); (ii) subtract an original depth image from the maximal depth value; (iii) save on 8-bits. The formal definition of this preprocessing pipeline is defined by

(1) $$D_{p}\left(i,j\right)=\mathrm{clip}\left(\left(\max_{a<w,b<h}D\left(a,b\right)\right)-D\left(i,j\right),\;0,\;255\right),$$

where w,h>0 denotes image width and height, D(i,j) is the raw depth value (saved on 16 bits) of the pixel in the *i*-th row and *j*-th column, while clip is a function that reduces the values of the first argument to the range defined by the second and third arguments. Although this operation clips the objects located further than 25.5 cm from the background level, in the proposed setting, such objects do not occur. Thus it is a valid lossless transformation.

Moreover, another preprocessing step was employed before feeding the data into neural networks. Both RGB and depth images were standardised using an empirical mean $\mu \in R^{w\times h\times c}$ and a standard deviation $\sigma \in R^{w\times h\times c}$ of the w×h px images, with *c* channels, from the training dataset, using

(2) $$\bar{x}=\frac{x-\mu }{\sigma },$$

where $x\in R^{w\times h\times c}$ denotes the exemplary image, and all operations are element-wise. That process is a standard procedure in the training of the neural networks, as without it, the tested networks would not be able to successfully generalise to unseen data.

Furthermore, we decided to test the results on depth data with an additional preprocessing step applied before normalisation. In this step, grayscale depth images were colored with the jet colormap. That method was successfully applied in other tasks requiring depth processing with the neural networks and proved its usefulness [28].

**Figure 4:**
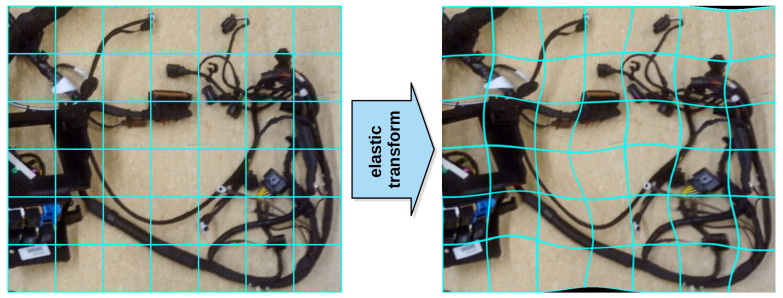
Sample effect of the elastic transform (**right**) on an example image (**left**). A blue grid is superimposed on the image to better illustrate the effect the transform has on the input image.

**Elastic transform**. In one of the experiments, data augmentation was complemented with the elastic transform operator to evaluate its impact on the performance. First introduced in the field of character and written text recognition [29], the transformation provides a good representation of the wire deformations that can occur in real-life conditions. An example image that has undergone an elastic transform is shown in Figure 4.

An essential element of the transform is the displacement grid, created by first randomly generating the displacement components for each pixel. Two random matrices with the same size as the image (w×h), filled with elements in the range of <−1, 1>, are generated, one for each component of the two-dimensional grid. They are then multiplied by the constant that sets the maximum displacement δ. Subsequently, the components are smoothed with a Gaussian filter with a variance of σ to ensure that neighbouring pixels have similar displacement. The final pixel values of the transformed image $I_{ET}$ are computed by picking the pixels from the source image *I* according to the computed displacement field—see Equation (3). Bilinear interpolation is used for fractional coordinates, and the settings were σ=50 and δ=50.

(3) $$\begin{array}{c} \Delta_{x}=G\left(\sigma \right)*\left(\delta Rand_{x}\left(w,h\right)\right)\\ \Delta_{y}=G\left(\sigma \right)*\left(\delta Rand_{y}\left(w,h\right)\right)\\ I_{ET}\left(j,k\right)=I\left(j+\Delta_{x}\left(j,k\right),k+\Delta_{y}\left(j,k\right)\right). \end{array}$$

**Inpainting**. One of the most common methods in the case of dealing with small datasets is pretraining the model on some pretext task to allow the neural network to learn representations of the image content that may be useful in the target task. In this paper, we trained an encoder–decoder architecture for an inpainting task on the images from our dataset. Input images were occluded with black rectangles of various sizes and positions, and the model was asked to retrieve the underlying image. By doing so, we conjecture that the encoder would learn important features for wiring harness representation, which may then be used for successful classification.

### 3.3. Evaluated Models

**Figure 5:**
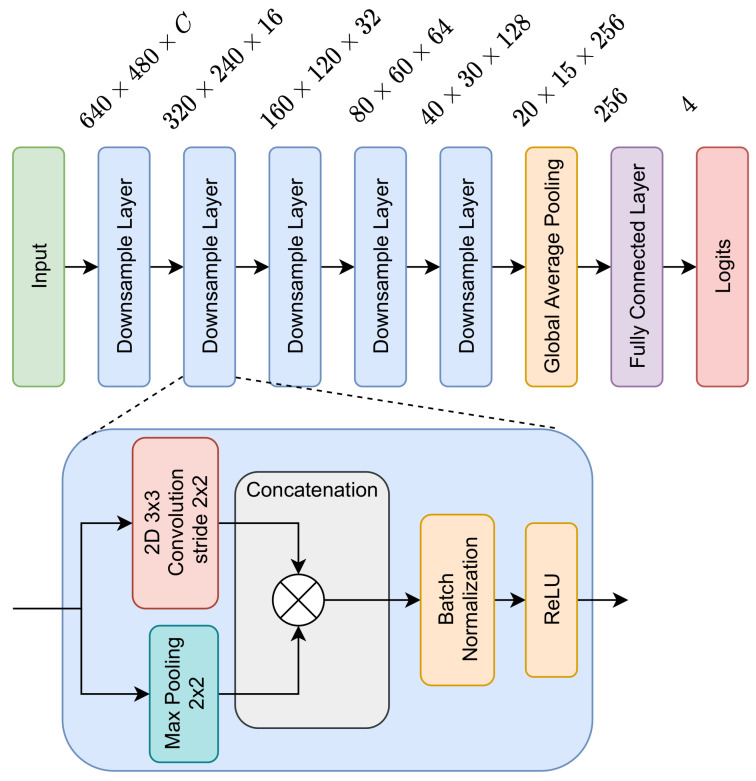
The architecture of the neural network used as a core of all models evaluated in the experiments. Numbers at the top of the figure define the size of the processed tensor. The main building block of this model, called the *Downsample Layer* [30], is depicted below.

As industrial applications are frequently required to operate under real-time or near real-time conditions, a decision was made to use a neural network architecture that provides a good balance between the achieved results as a base for the construction of the model. In our experiments, we tested several neural network models to solve the task of wiring harness classification. All of those networks shared the same core architecture, which is depicted in Figure 5. The main building block of this neural network is the *Downsample Layer* from the ERFNet model [30], which achieved real-time performance in the task of semantic segmentation on the Cityscapes dataset [31]. After a few *Downsample Layers*, the data are passed through the *Global Average Pooling Layer*. Finally, a vector of four logits (by a logit, we mean the logarithm of the probability assigned to the given class) is determined with the use of a fully-connected layer.

Since the gathered data consist of two modalities (RGB and Depth), several different models, exploiting the network mentioned above, were proposed:

- RGB—a model that takes as an input an RGB image only;
- Depth—a model that takes as an input an 8-bit depth image only;
- Depth Jet—a model that takes as an input a 24-bit depth image colored with the jet color-map;
- RGBD—a pair of RGB and Depth models with the *logits weighting*;
- RGBD jet—a pair of RGB and Depth Jet models with the *logits weighting*;
- RGBD early fusion—a model that takes as an input an RGB image concatenated with a depth image.

Moreover, for the comparison, we also used a sum of logits of two models to evaluate whether there is any gain from such a late prediction fusion [32]. We examined two instances of this type of model: (i) RGB and Depth (denoted as *RGB + Depth*) and (ii) RGB and Depth Jet (*RGB + Depth Jet*). Furthermore, we analysed the impact of two popular methods for dealing with small datasets: **D**ata **A**ugmentation (RGB + Depth + **DA**) and pretraining on the pretext task—**IN**painting (RGB + Depth + **IN**).

The aforementioned *logits weighting* is a procedure applied to logits obtained from two models, where logits from the first model $l_{1}$ are weighted with the ones from the other model $l_{2}$. The weighting formula is as follows:

(4) $$l=wl_{1}+\left(1-w\right)l_{2},$$

where w∈[0;1] is a trainable parameter, based on which one can see which modality is considered by the network as more reliable.

After all of these procedures, at the very end, a softmax function σ, defined by

(5) $$\sigma_{i}\left(x\right)=\frac{e^{x_{i}}}{\sum_{j=0}^{N}e^{x_{j}}}\;\mathrm{for}\;\mathrm{all}\;i\in \left\{1,2,\ldots ,N\right\},$$

where *N* is a number of classes, is applied to the logits (denoted in (5) as $x_{i}$) to obtain valid class probabilities.

### 3.4. Class-Based and Class-Agnostic Saliency Map Generation

To analyse the basis of the decision made by our neural network models, we want to verify which regions of an image attracted the greatest attention of the classifier. To achieve this, we use two methods: class-based and class-agnostic saliency map generation.

The first algorithm was introduced in [27]. It uses the feature maps from the last layer before the *Global Average Pooling* and multiplies it by the weight matrix from the fully-connected layer associated with one of the considered classes to obtain the saliency map for this specific class, which can be written as follows

(6) $$S_{c}\left(i,j\right)=\sum_{k=0}^{256}w_{k,c}F\left(i,j,k\right),$$

where $S_{c}\left(i,j\right)$ is a pixel in the *i*-th row and *j*-th column of the saliency map for class *c*, $w_{k,c}$ is a weight between the *k*-th feature and class *c*, obtained form the weight matrix in the fully-connected layer, while F denotes the last feature map. This method provides information about which parts of the image are encouraging and discouraging the final classifier to choose a given class.

The second algorithm is our adaptation of the *class activation mapping* method introduced in [27]. Similar to [27], we took activations from the last convolutional layer of each neural network and performed a summation along the feature dimension, but without weighting the features by the feature-class weights from the fully-connected layer, which can be written as follows:

(7) $$S\left(i,j\right)=\sum_{k=0}^{256}F\left(i,j,k\right).$$

Both procedures provide the smaller version of an input image (20×15 px; however for visualisation purposes, we resize it to the shape of the original image). In our modified algorithm, larger values suggest that there are some features that are crucial for the choice of a final class in their place. In contrast, smaller ones indicate regions without a significant amount of information essential for the classification process. As a result, we obtain a map, which gives us some insight into which parts of an image are salient for the classification. In the original CAM algorithm, large values indicate the parts of the image that encourage the classifier to choose a given class. In contrast, the lower values suggest regions that discourage the choice of a particular class.

## 4. Experiments

The experimental evaluation of neural network models in the task of classification of the branches of the wire harness is divided into two parts. First, we analysed the accuracy achieved by the models, whereas in the second part, we focused on analysing the model’s reliability.

### 4.1. Model Accuracy Evaluation

To evaluate the accuracy of our neural network models, we employed the 3-fold cross-validation. Each classifier was implemented in Tensorflow [33] and trained for 300 epochs on the training part of the data (2 out of 3 folds), which took about 1 hour per model on a RTX 3080 GPU. We used mini-batches of size 16, and the Adam optimiser [34] with a learning rate equal to $10^{-4}$. For each model, the weights that achieved the highest accuracy on the validation dataset were used for evaluation. The results for all tested models are reported in Table 1. We reported the accuracies only, as the running times of the considered models were relatively low and comparable, as they were equal to approximately 4 ms for all models, except those which utilise the late fusion, as their inference time was equal to 8 ms because they have to evaluate two sub-models.

The obtained results show that the difference between models utilising Depth and RGB data is minor. However, the RGB data leads to slightly better accuracy. Moreover, the best results were obtained for the models that processed the data from both modalities, regardless of how the data fusion was performed. Surprisingly, the worst overall results were obtained by models that used the depth data preprocessed with the jet coloring scheme, which performed the worst for the split I, III/II.

The best 3-fold average accuracy was achieved by models utilising the sum of predictions of two models—so-called late fusion (due to that, this model was also chosen for more extensive tests with data augmentation and pretraining). The RGB + Depth classifier obtained 94.01% mean accuracy on the validation datasets, whereas the RGB + Depth jet obtained the highest mean accuracy of 96.76% on the training datasets. We observe that models with the highest 3-fold average accuracy had the smallest variance of results between folds, which, together with relatively high accuracy, allowed for the outperformance of the rest of the models. For other classifiers, the results differ notably between dataset splits, for example, the RGBD model achieved impressive results on validation datasets on two folds (94.5% and 99.5%). However, the third one performed the worst (84.58%). That, in turn, affected the average results significantly.

Besides the default models, we tested two methods for dealing with small datasets and analysed whether they can improve the results obtained by the basic models. While using data augmentation allowed us to achieve a much better performance than training only on the raw dataset, that is, 100% on training and 95.5% on validation sets on average, pretraining on the inpainting pretext task led to a significant drop in the performance. This decrease in the classification performance may be caused by the pretext training, which encouraged the model to pay much attention to the wiring harness configuration, which is relevant for reconstruction and inpainting but is irrelevant for the classification task. Simultaneously, the data augmentation with the use of the elastic transform allowed the model to neglect the exact configuration of the wiring harness branch and instead focus on some key features that help differentiate between classes. Interestingly, the model pre-trained on the inpainting pretext task did not achieve the results of the models trained from scratch. This may be because a neural network trained on a pretext task fell into some local minimum during the training because the pretext task moved neural network weights from a random place on the optimisation manifold into some adverse neighbourhood.

Moreover, we observe that almost all models (except RGBD jet and Depth jet) showed the most significant differences between accuracy on training and validation datasets for the first split (I, II/III). Those differences were usually minor for the other two splits (about 1 to 4 percentage points). This could suggest that reasoning about fold III of the dataset, using I and II for training, was the most challenging task for our models.

### 4.2. Analysis of the Models Reliability

**Figure 6:**
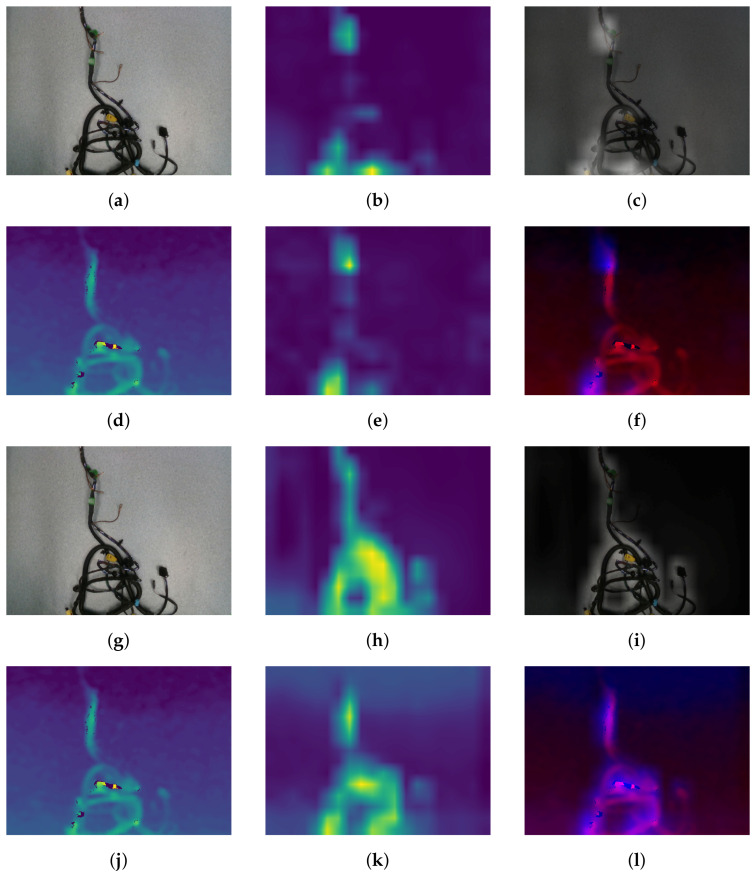
Class agnostic saliency maps generation method applied to the RGB + Depth (1st and 2nd row) and RGB + Depth + DA (3rd and 4th rows) models on the image of wiring harness from the validation dataset (fold I, III/II). Images from first column represents the input data: (**a**,**g**) —RGB image, (**d**,**j**)—depth data. In the second column, there are generated saliency maps, computed with the method described in Section 3.4, while in the last column, the input images with saliency maps superimposed on them. For the depth images, we changed the color scheme in the last column, such that the depth is represented by the intensity on the red channel, while the saliency is represented in the blue channel.

Figure 6 shows the class-agnostic saliency maps obtained for both RGB and depth data by the RGB + Depth model trained from the plain dataset and the same model, but trained on the augmented data. Both neural network models for both modalities ignored the background and focused on the wiring harness. Interestingly, while the model without data augmentation focused only on some specific parts of the branch—that is, some connectors and characteristic elements of the branch—the addition of the data augmentation encouraged the model to pay attention to the whole wiring harness, which allowed it to achieve a higher accuracy. Focusing only on some tiny parts of the image suggests that the model overfits to some parts of the harness crucial for the decision, while neglecting the rest. This may lead to the mistakes caused, for example, by the self-occlusions, which are frequent in the entangled harness. In turn, by performing elastic transformations of inputs, the neural network learned to find the whole harness in the image and use the features gained along with the entire harness, which is invariant to the elastic transformation, which is typical for the real configurations of the wiring harness.

**Figure 7:**
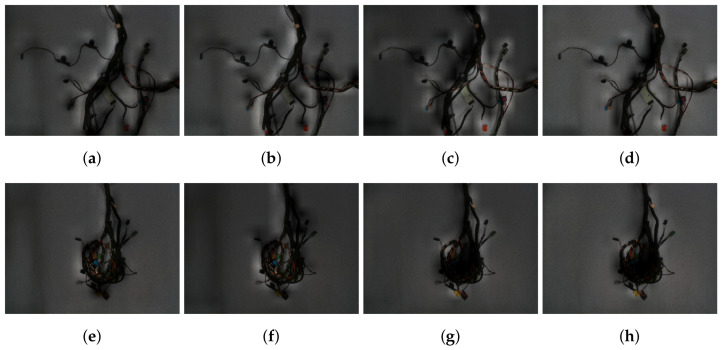
Class activation saliency maps, superimposed on the 2 different RGB images, conditioned on 4 classes (*branch 1*–*4* from left to right). Top row (**a**–**d**) contains the saliency maps obtained for the successful classification (ground truth and predicted class: *branch 3*—(**c**)), while in the bottom row (**e**–**h**) for a failure case (ground truth class: *branch 3*—(**g**), predicted class: *branch 4*—(**h**)). For the successful scenario neural network focuses its attention to the specific parts of the *branch 3* (**c**), while for other classes (**a**,**b**,**d**) it can not find relevant parts of the branch. In the failure case, tangled branch occludes the parts of the branch important for distinguishing between the classes. Neural network finds an evidence both for *branch 3*—(**g**) and *branch 4*—(**h**) and it is unable to correctly decide which is more likely.

Moreover, we analysed the saliency maps conditioned on all classes, depicted in Figure 7, to determine which parts of the image are interesting to the neural network in terms of choosing a specific class. In our experiment, we used two exemplary dataset elements (only RGB images) conditioned on four classes (from left to right) to illustrate the regions important to each class in the case of successful classification (1st row (a–d), ground truth and predicted class: *branch 3*—(c)) and misclassification (2nd row (e–h), ground truth class: *branch 3*—(g), predicted class: *branch 4*—(h)). In the first case, it is visible that, while all classes consider different parts of the branch important, the actual class prediction is supported by the perfectly visible electrical connectors and some specific parts of the branch such as the green plate (see Figure 7c). However, in the second case, most of the specific connectors and parts of the branch are not visible to the system, causing problems with the classification. Even though two yellow connectors are visible, they are a stronger indicator of the *branch 4* class than for *branch 3*, in the situation where other specific parts are not present.

**Figure 8:**
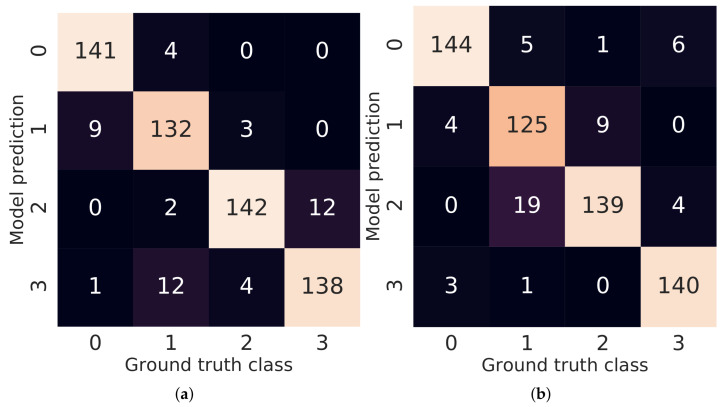
Confusion matrices for RGB (**a**) and Depth (**b**) models.

The confusion matrices for both RGB and Depth models are presented in Figure 8. The analysis of those matrices allow us to speculate why the RGB + Depth model achieved the best average accuracy in a 3-fold cross-validation on the validation dataset. The RGB model makes different types of misclassification in comparison with the Depth model. Those differences enable the joint RGB + Depth model to level the errors of sub-models by reconciling the final prediction using the beliefs from both models.

### 4.3. Analysis of the Dataset

**Figure 9:**
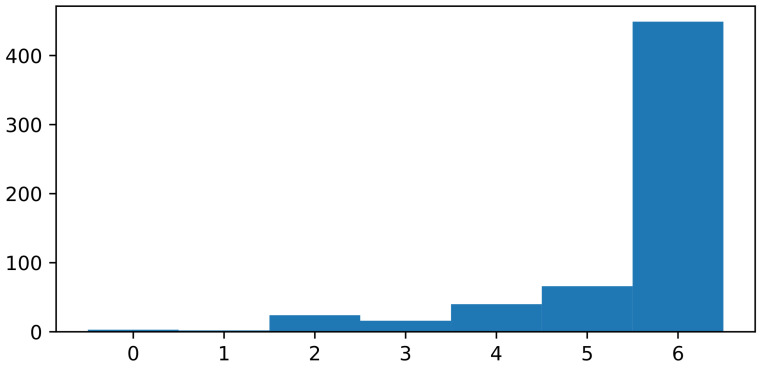
The number of instances from the dataset, for which a specific number of models (listed in Section 3.3) predicted the right class.

In Figure 9, we presented the results of the introduced dataset’s consistency test using the models from Section 3.3. We observe that the number of examples in the dataset, for which all of the models were unable to predict the right class, is very small. That observation leads to the conclusion that there are almost no examples in the dataset that are so different from the others that they mislead all of the evaluated models. Moreover, it suggests that the ensemble of models could lead to an even better classifier.

## 5. Conclusions

In our work, we introduced a new dataset of 600 RGBD images of wiring harness branches from the *Volkswagen Caddy* cockpit. We trained several neural network models on images from the dataset to classify branches of the wiring harness. Moreover, we performed a reliability analysis of our neural networks and also the dataset consistency test.

The obtained results showed that it is possible to train a neural network model, even without large amounts of data. Our model, trained on a raw dataset, achieved 94% accuracy on average on the 3-fold cross-validation. The best performing model—RGB + Depth—used predictions from two independent models—RGB and Depth—to produce the probability distribution of classes given an RGBD image. The analysis of the result of the RGB + Depth model and its confusion matrices for those models separately may suggest that the RGB and Depth modalities convey slightly different information and should complement each other in the decision process. However, those modalities do not have to be processed jointly from the early stage (RGBD early fusion model) nor trained jointly (RGBD model). Moreover, we showed that augmenting the data using elastic transform, which simulates even more different plausible configurations of the harness, leads to superior results.

The analysis of the reliability of the networks showed that the model trained on the raw dataset for both modalities focuses more on electrical connectors, which are very distinctive. That behavior suggests that the models ignore the appearance of the wire harness branch as a whole, but instead try to find some specific connectors. Even if this approach seems reasonable, it can fail in the case of the self-occlusions of the most important connectors. The use of the elastic transform to augment the data not only allowed us to obtain a better accuracy but also changed the way the network perceives the harness branch. In this case, the neural network focuses on the whole branch, neglecting the background only. This does not mean that the network does not pay attention to the connectors, but it is much more reliable because it can decide even in some cases when the connectors are not enough to predict the right class.

The statistical analysis of the dataset’s consistency showed that it is almost free from the examples, which are so different from the others that it makes them impossible to classify using the data from the other folds.

The study presented in this paper focused on the classification of the branches of the wiring harness, which is the first step towards the perception system, which enables the precise robotic manipulation of the wiring harness. A good quality, explainable, top-level overview of the wiring harness branch location, which can be derived from the classification results, is a valuable basis for a more complex processing pipeline. As a next step, we plan to focus on wiring harness segmentation, which will help the robotic platform precisely differentiate and localise individual wires of the wiring harness branches in the images.

The following abbreviations are used in this manuscript:

## Figures and Tables

**Table 1:** The accuracy [%] of the considered models in the task of wiring harness classification for the 3-fold cross-validation.

Dataset Split	I, II/III		II, III/I		I, III/II		3-Fold Average	
**Model**	**Training**	**Validation**	**Training**	**Validation**	**Training**	**Validation**	**Training**	**Validation**
RGB	**99.0**	88.1	94.3	92.5	92.0	96.0	95.1 ± 3.6	92.2 ± 4.0
Depth	93.3	86.6	95.8	93.0	93.8	94.5	94.3 ± 1.3	91.4 ± 4.2
Depth jet	96.3	90.1	**97.5**	**94.5**	93.5	81.0	95.8 ± 2.0	88.5 ± 6.9
RGBD	91.3	84.6	95.5	**94.5**	**99.0**	**99.5**	95.3 ± 3.9	92.9 ± 7.6
RGBD jet	96.3	88.6	95.0	92.0	81.6	67.5	90.9 ± 8.2	82.7 ± 13.3
RGBD early fusion	96.5	90.1	96.8	92.5	95.0	98.0	96.1 ± 0.9	93.5 ± 4.0
RGB + Depth	95.5	**91.5**	96.5	94.0	95.8	96.5	95.9 ± 0.5	**94 ± 2.5**
RGB + Depth jet	96.3	89.6	**97.5**	93.5	96.5	94.0	**96.8 ± 0.7**	92.4 ± 2.4
RGB + Depth + IN	69.0	63.7	64.8	75.0	69.8	77.5	67.9 ± 2.2	72.1 ± 6.0
RGB + Depth + DA	**100.0**	**92.5**	**100.0**	**98.0**	**100.0**	**98.0**	**100.0 ± 0.0**	**95.5 ± 2.3**

## Data Availability

Dataset is available at https://chmura.put.poznan.pl/s/XIiZgNfCMCuj1bY/download, accessed on 22 June 2021.
